# Supplementary material for: Realizing long-cycling all-solid-state Li-In||TiS2 batteries using Li6+xMxAs1-xS5I (M=Si, Sn) sulfide solid electrolytes
Source: Nat Commun. 2023 Jul 10;14:4077. doi: 10.1038/s41467-023-39686-w (PMC10333182; doi:10.1038/s41467-023-39686-w)
Supplement: Supplementary file 1 — Supplementary Information [file 41467_2023_39686_MOESM1_ESM.docx]

**Supplementary Information**

**Realizing long-cycling all-solid-state Li-In||TiS_2_ batteries using Li_6+x_M_x_As_1-x_S_5_I (M=Si, Sn) sulfide solid electrolytes**

Pushun Lu^1,2^, Yu Xia^3^, Guochen Sun^1,2^, Dengxu Wu^1,2^, Siyuan Wu^1,2^, Wenlin Yan^1,2^, Xiang Zhu^4,5^, Jiaze Lu^1,2^, Quanhai Niu^5^, Shaochen Shi^3^, Zhengju Sha^3^, Liquan Chen^1,2,5,6^, Hong Li^1,2,4,5,6^*, Fan Wu^1,2,4,5,6^*

**Affiliations:**

^1^Institute of Physics, Chinese Academy of Sciences; Beijing, 100190, China.

^2^School of Physical Sciences, University of Chinese Academy of Sciences; Beijing, 100049, China.

^3^Beijing ByteDance Technology Co Ltd; Beijing, 100098, China.

^4^Nano Science and Technology Institute, University of Science and Technology of China; Suzhou, 215123, China.

^5^Tianmu Lake Institute of Advanced Energy Storage Technologies; Liyang 213300, Jiangsu, China.

^6^Yangtze River Delta Physics Research Center; Liyang 213300, Jiangsu, China.

*Corresponding author. Email: hli@ iphy.ac.cn (H.L.), fwu@iphy.ac.cn (F. W.)

**Supplementary Figure 1.** The XRD patterns of interested “LASI-30Sn” samples after sintering at different temperatures (550, 575, and 600 ℃).

**Supplementary Figure 2.** The XRD patterns of interested “LASI-80Si” samples after sintering at different temperatures (550, 575, and 600 ℃).


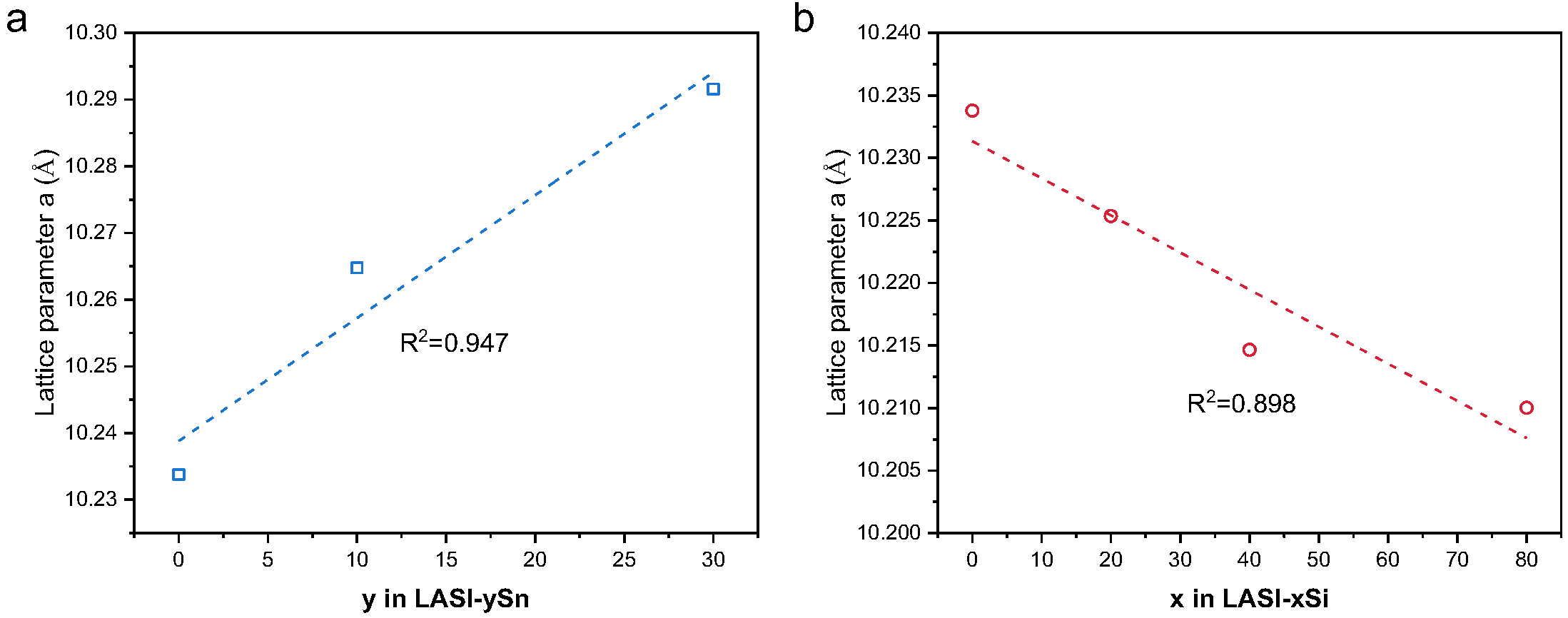
 **Supplementary Figure 3.** Lattice parameter of (a) LASI-ySn (y = 0, 10, 30) and (b) LASI-xSi (x = 0, 20, 40, 80) as a function of Sn and Si substitution content, respectively.


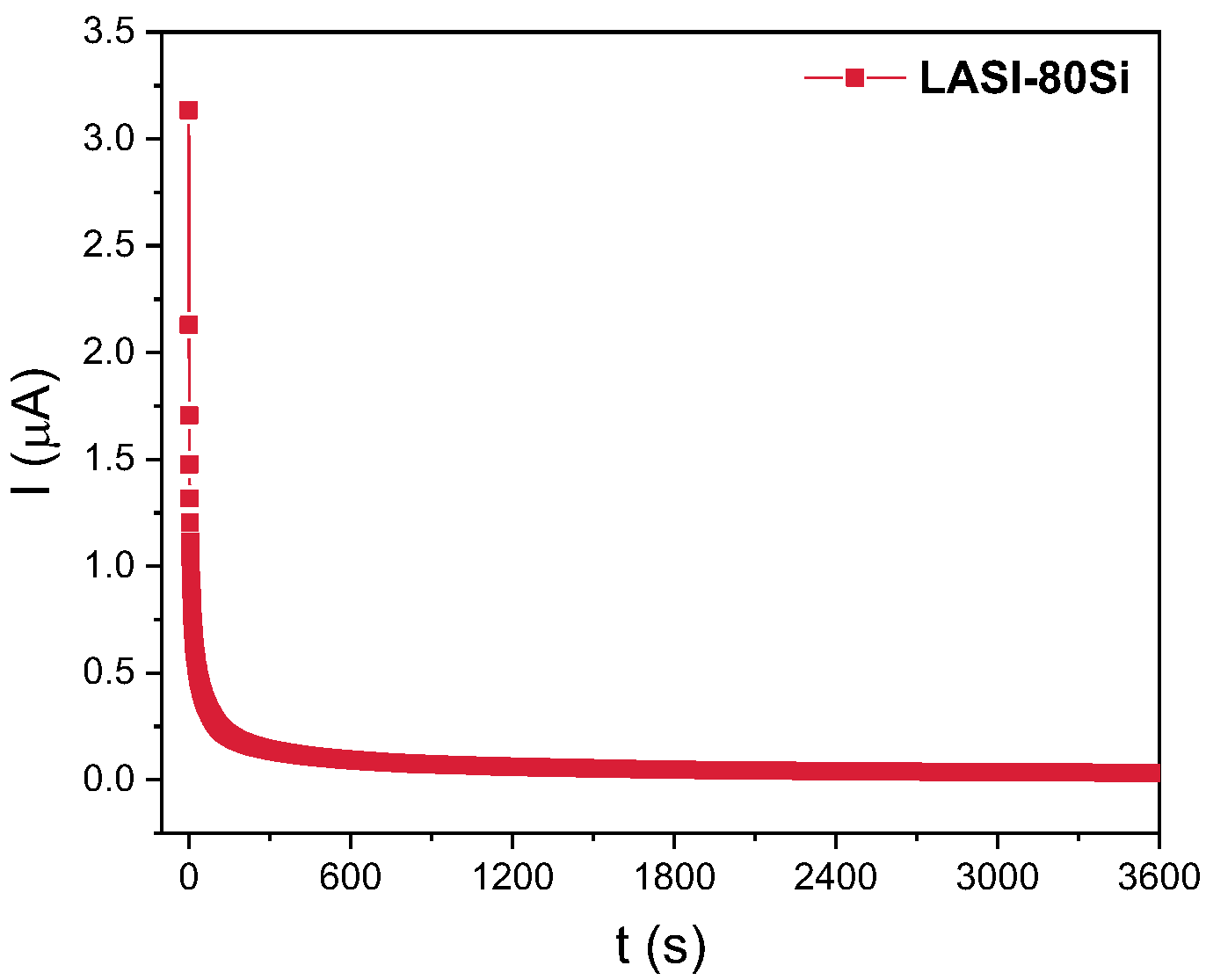


**Supplementary Figure 4.** Direct current polarization of LASI-80Si, which is tested in a cell configuration (SS|LASI-80Si|SS) with stainless steel (SS) as electrode and solid electrolyte (SE) as separator at 25 ℃.

**Supplementary Figure 5.** Rietveld refinement of powder X-ray diffraction data of LASI-80Si electrolyte.


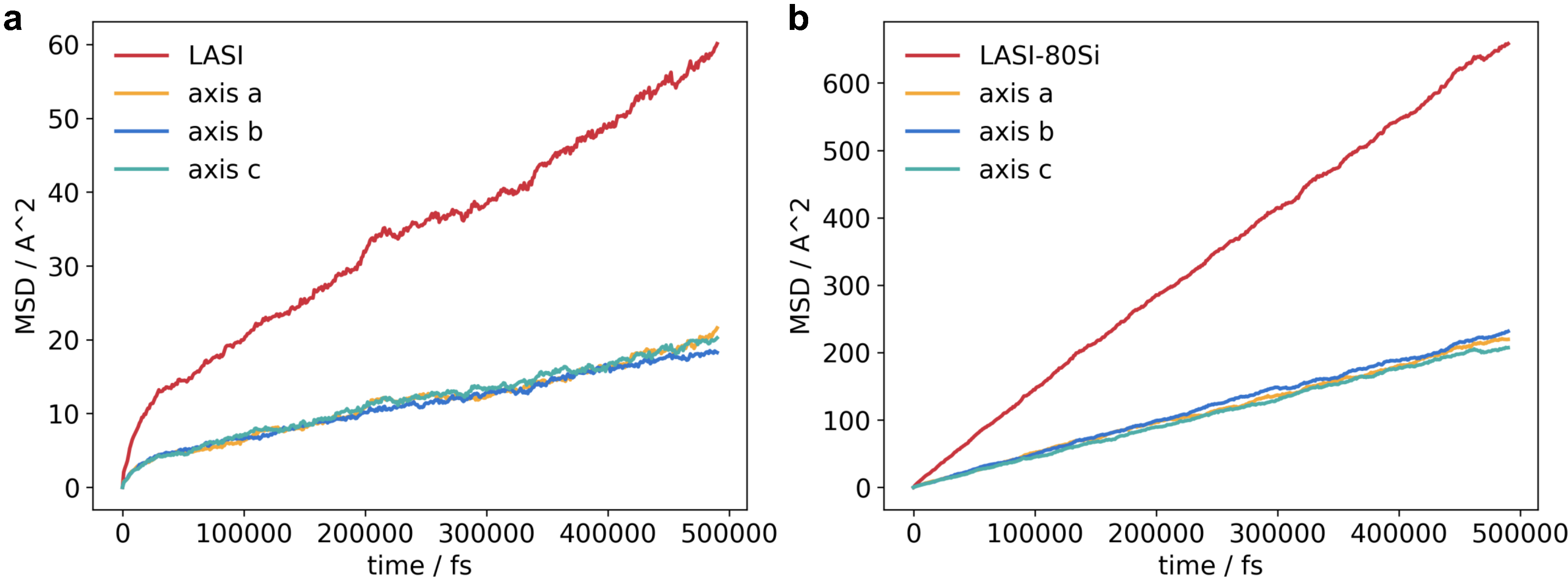


**Supplementary Figure 6.** Mean square displacement (MSD) of (a) LASI and (b) LASI-80Si within 0.5 ns at 800K.


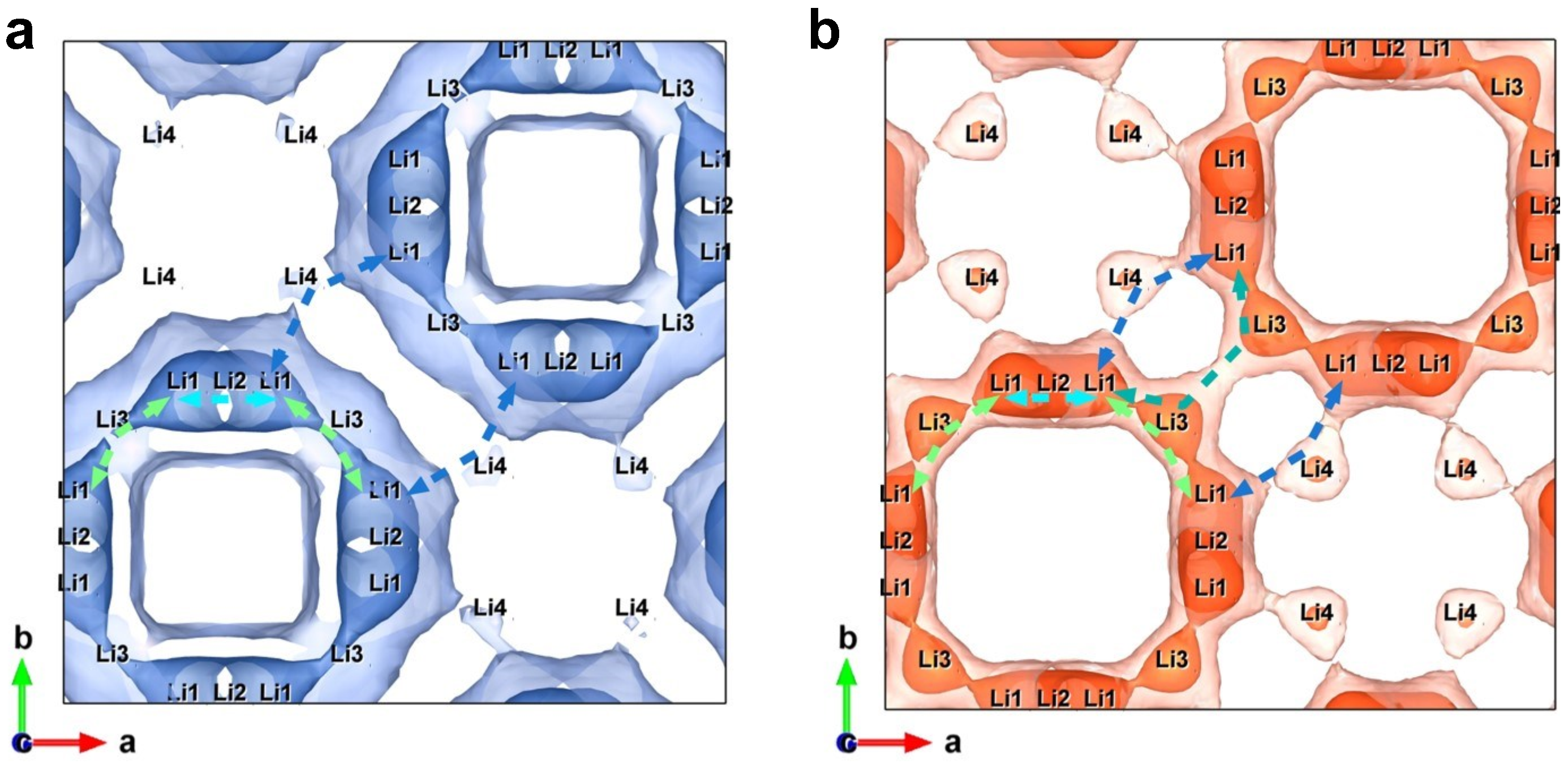


**Supplementary Figure 7.** 2D Li-ion probability density distribution of (a) LASI at 1000 K and (b) LASI-80Si at 400 K viewed from [001] direction. The doublet jump pathway (Li1(48h)-Li2(24g)-Li1(48h)), intra-cage jump pathway (Li1(48h)-Li3(48h)-Li1(48h), inter-cage jump pathway (Li1(48h)-Li4(16e)-Li1(48h) and new inter-cage jump pathway (Li3(48h)-Li3(48h)) are represented by the bidirectional arrows with light blue, light green, dark blue and dark green color, respectively.


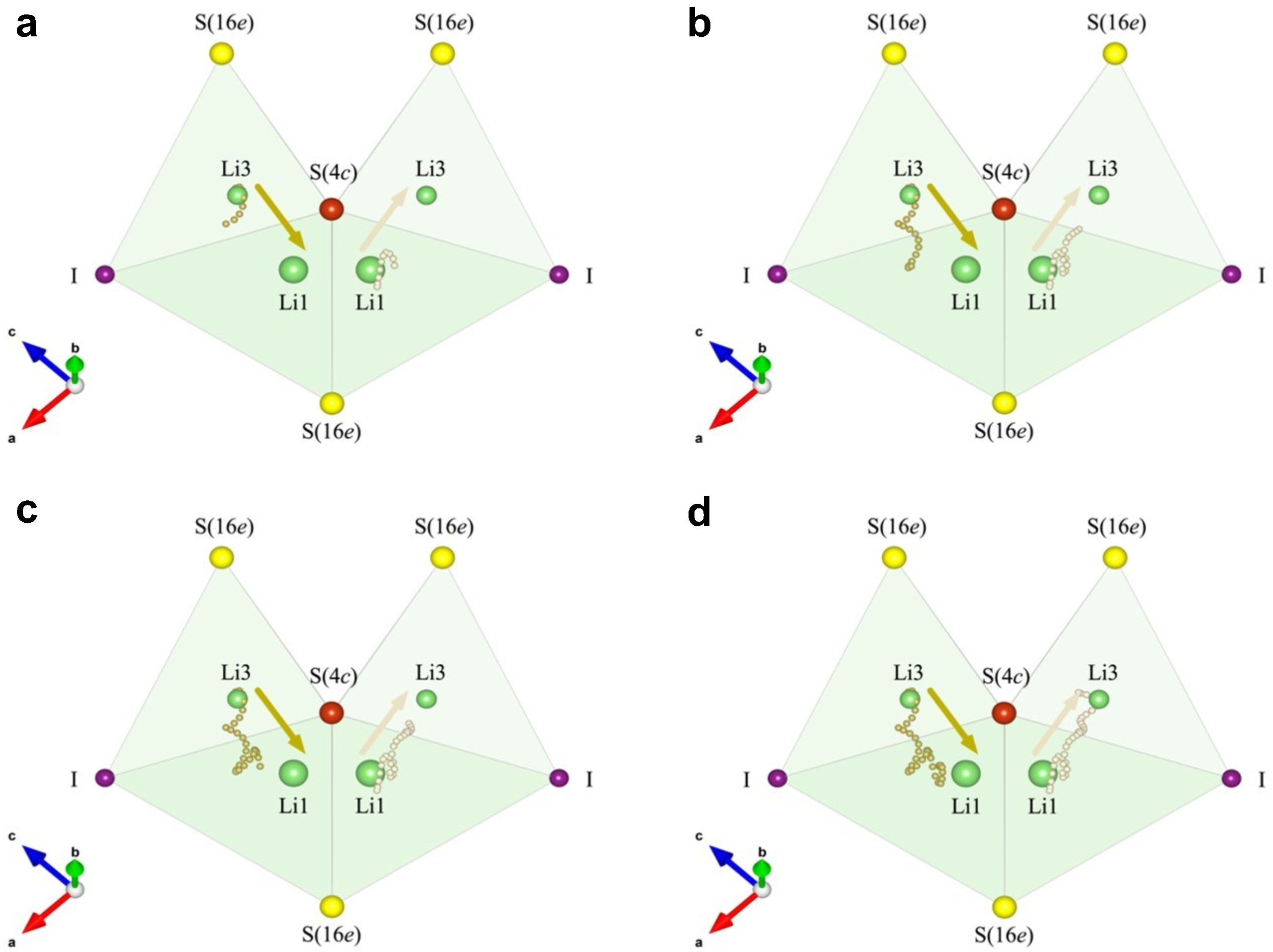


**Supplementary Figure 8.** The time-series trajectories of Li-ion migration inside the cage for LASI-80Si at (a) 100 fs, (b) 200 fs, (c) 300 fs and (d) 400 fs.


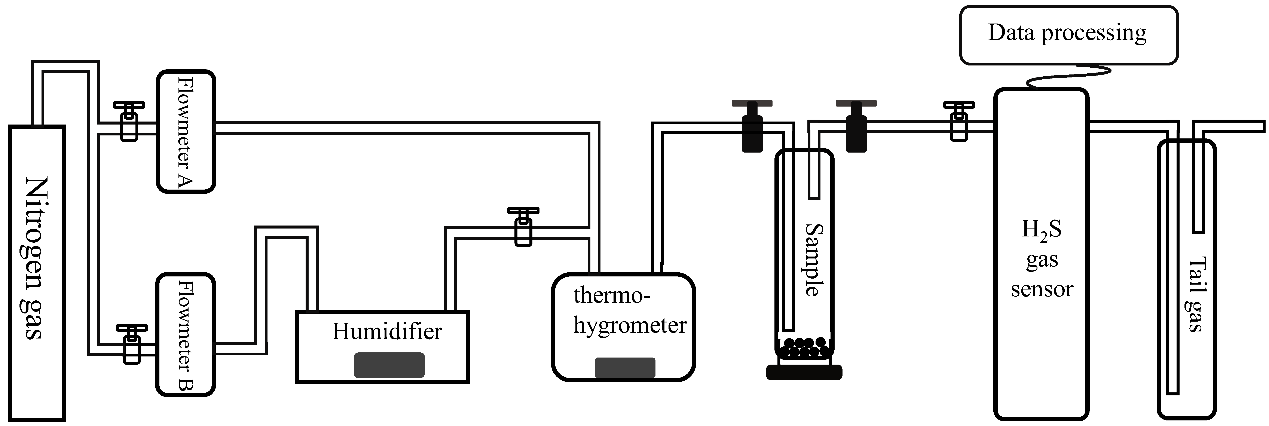


**Supplementary Figure 9.** Schematic of the in-house developed detection system for the measurement of H_2_S gas.


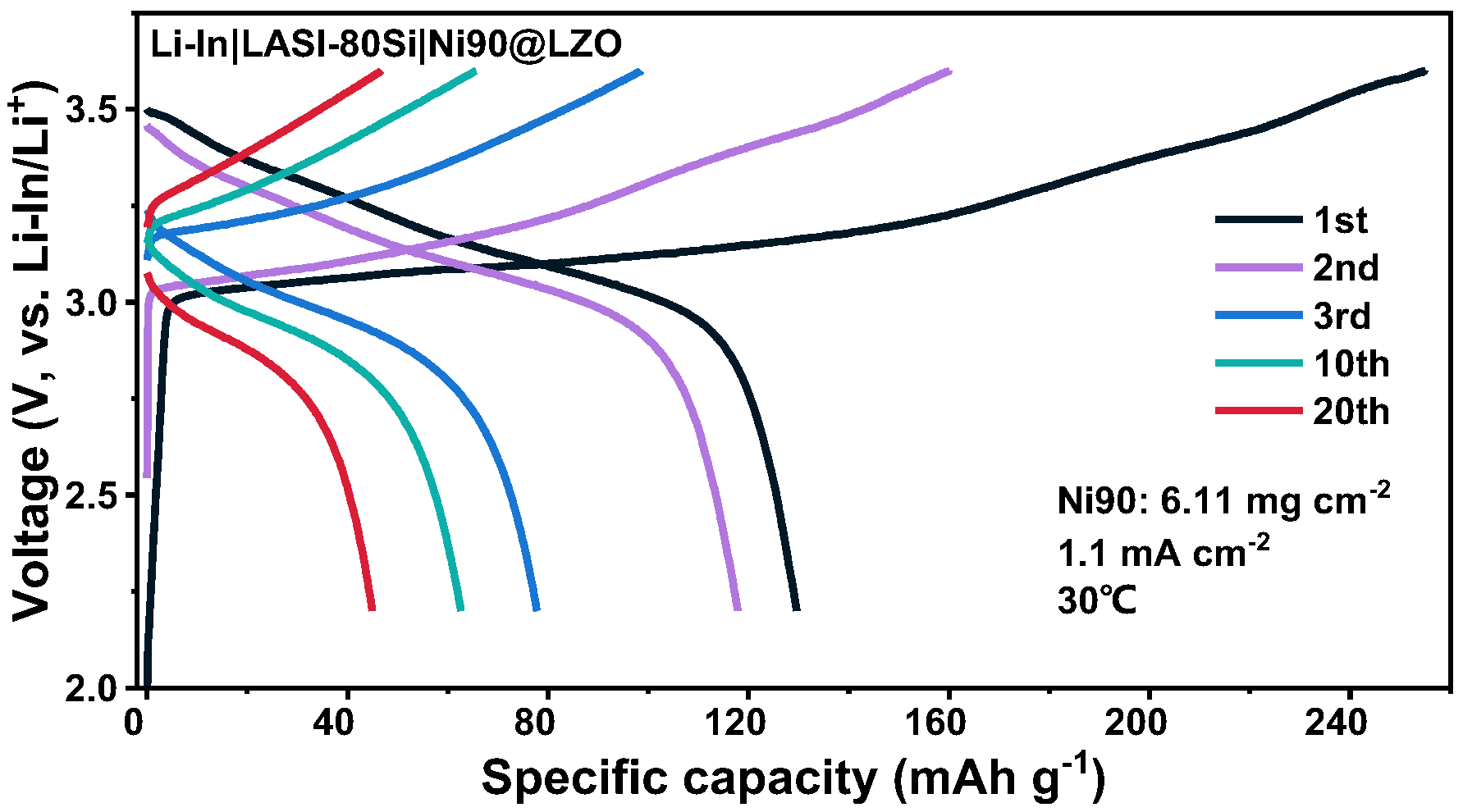


**Supplementary Figure 10.** The charge-discharge profiles of Li-In|LASI-80Si|Ni90@LZO all-solid-state battery at 30℃. Note that the current density of the first-two cycles is 0.22 mA cm^-2^ and that of subsequent cycles is 1.1 mA cm^-2^.


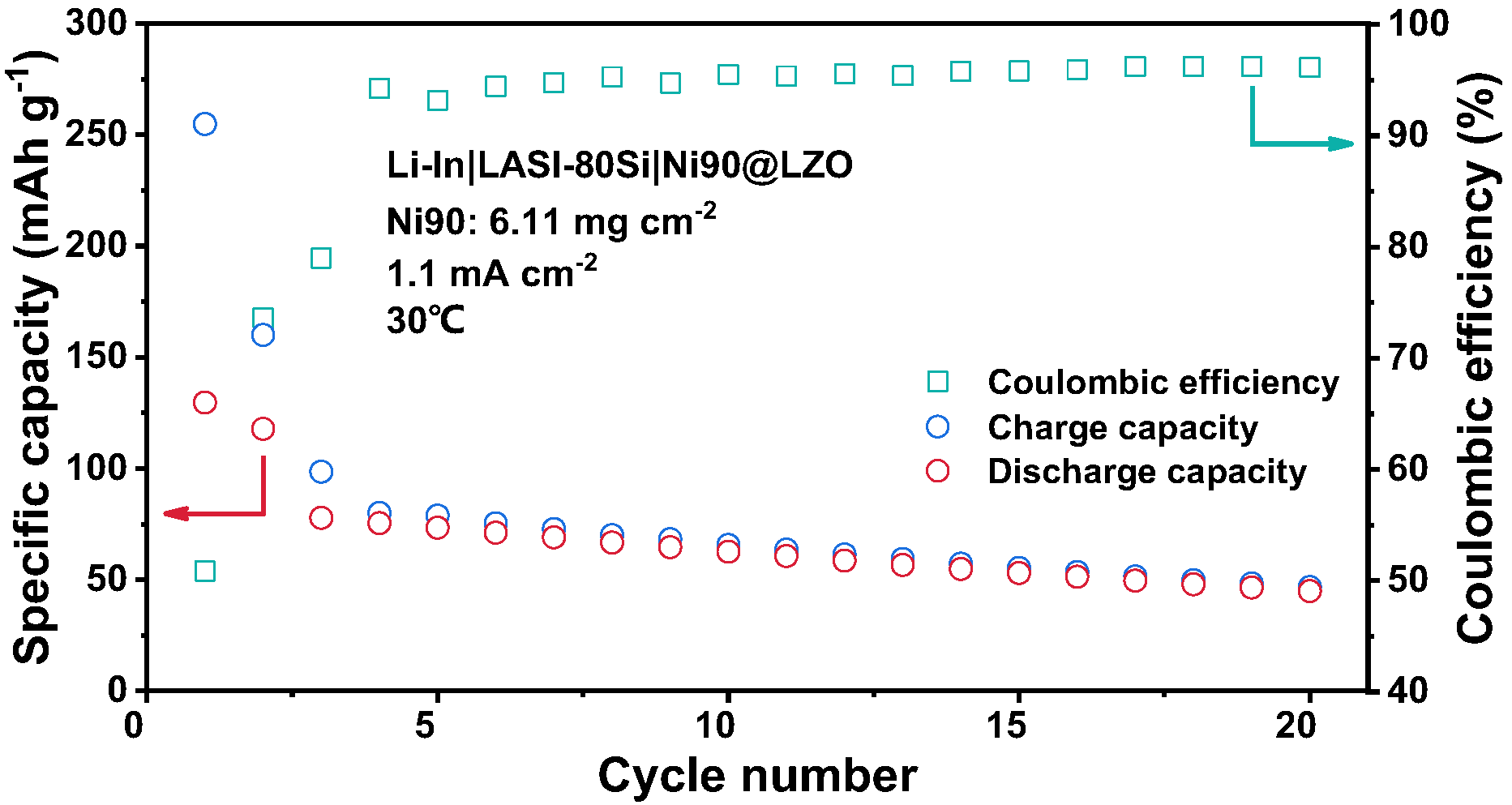


**Supplementary Figure 11.** The long-term cyclability of Li-In|LASI-80Si|Ni90@LZO all-solid-state battery at 30℃. Note that the current density of the first-two cycles is 0.22 mA cm^-2^ and that of subsequent cycles is 1.1 mA cm^-2^.


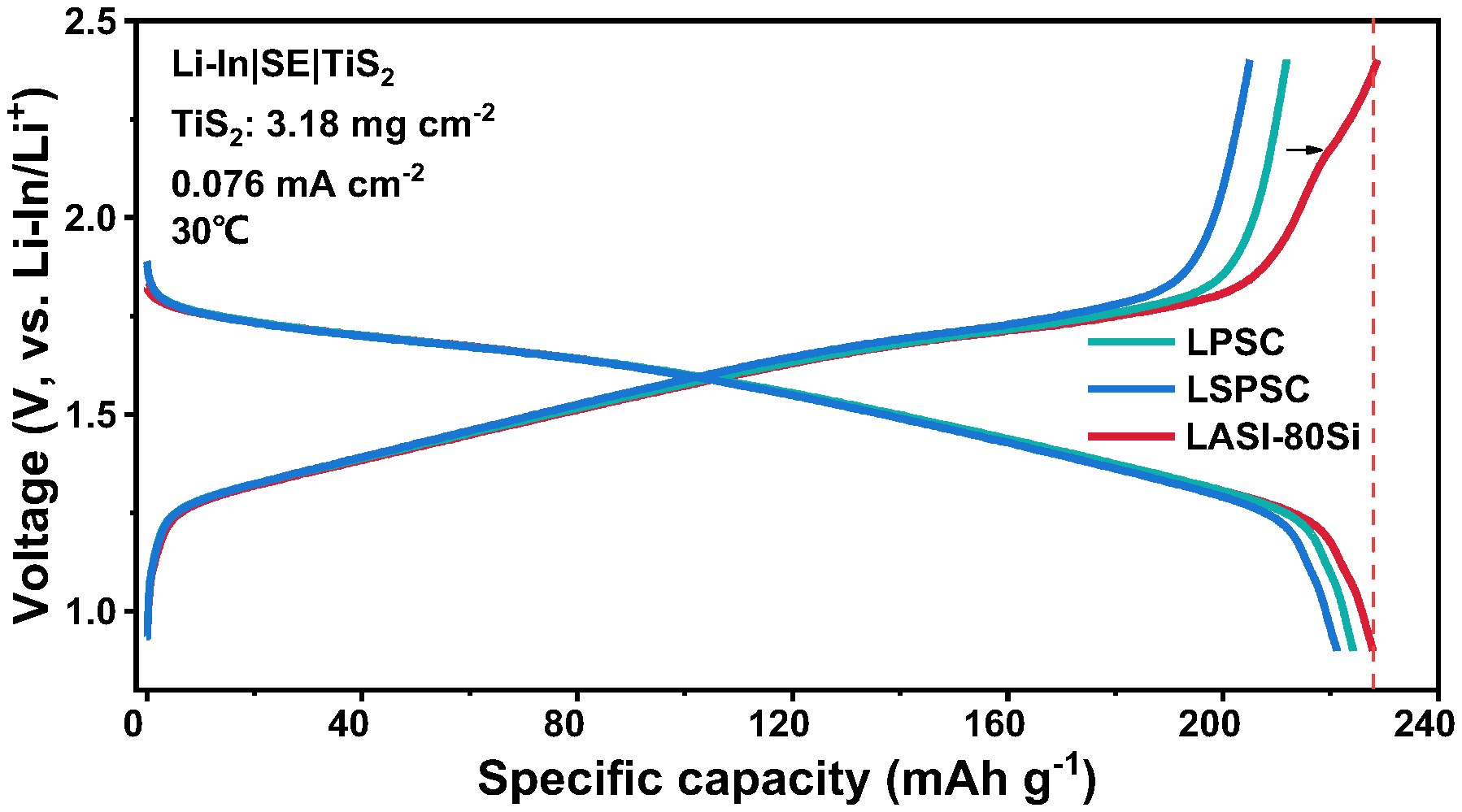


**Supplementary Figure 12.** The first-cycle discharge-charge profiles of for Li-In|SE|TiS_2_ ASSBs with LPSC, LSPSC and LASI-80Si sulfide SEs at 30℃ and 0.076 mA cm^-2^ current rate.


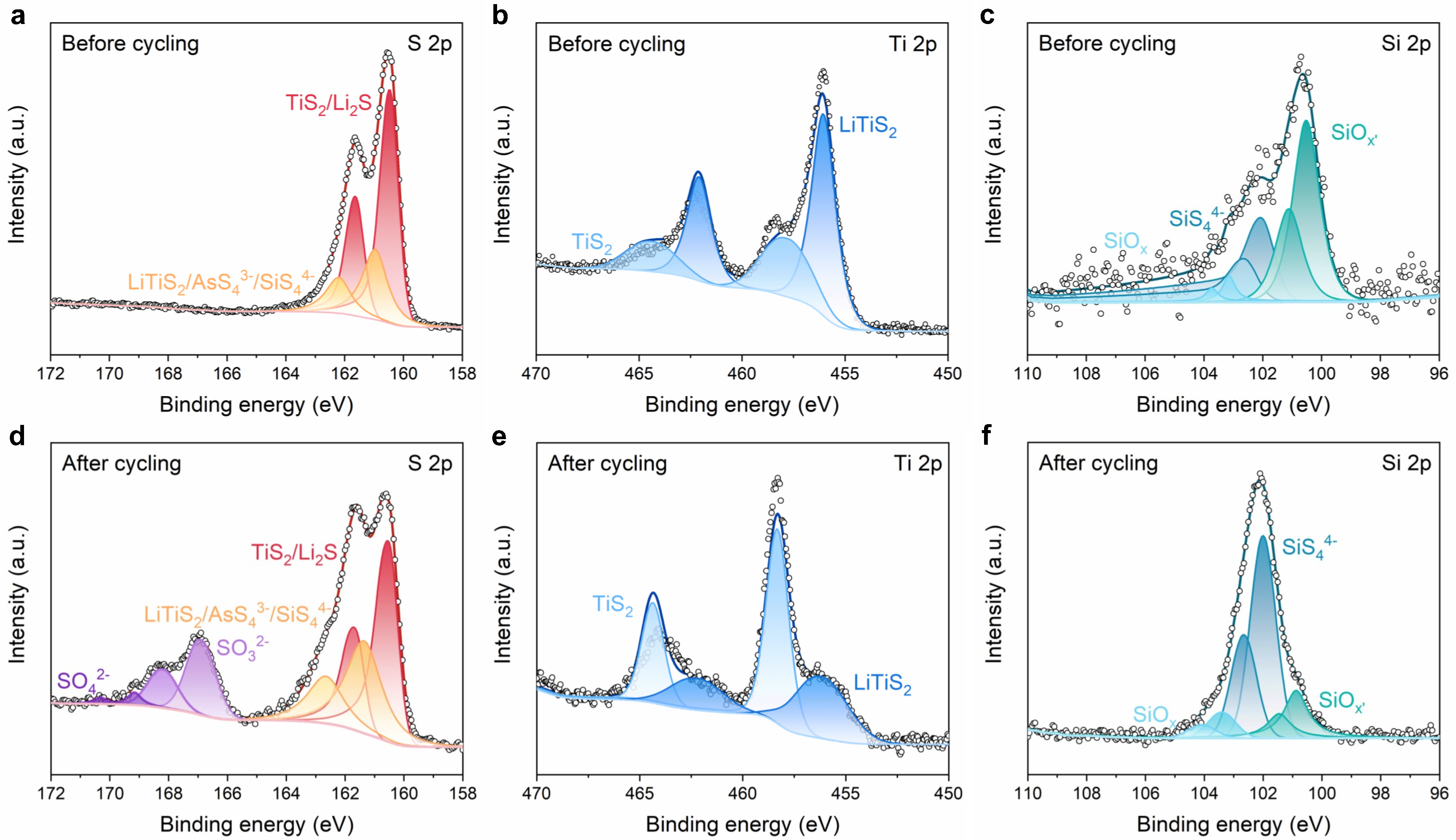


**Supplementary Figure 13.** XPS spectra of (A, B) S 2p, (C, D) Ti 2p and (E, F) Si 2p for TiS_2_/LASI-80Si composite electrode in Li-In|LSPSC|TiS_2_ cell before and after cycling (fully charged state) for 1000 cycles at 0.076 mA cm^-2^ and 30℃.


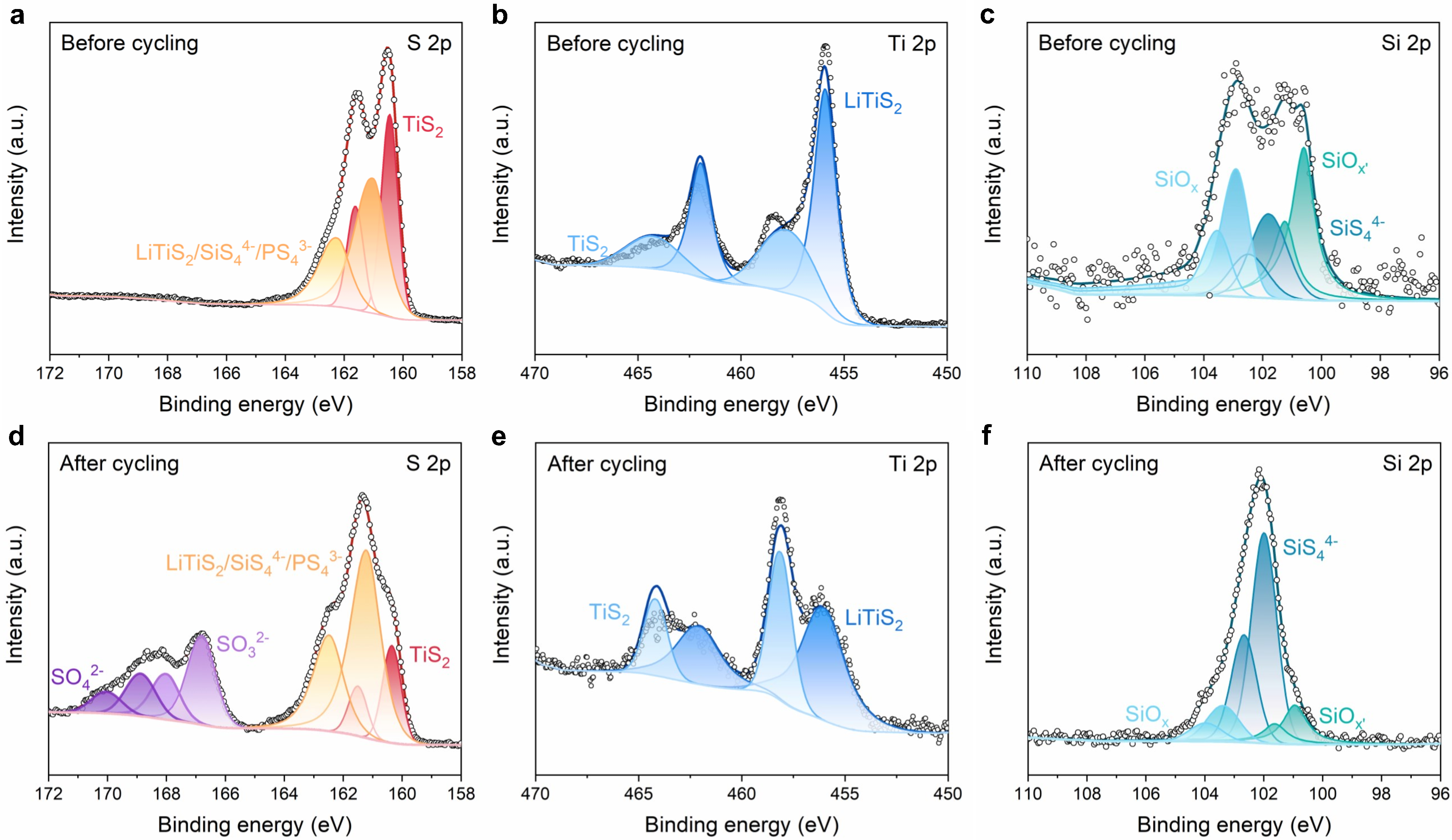


**Supplementary Figure 14.** XPS spectra of (A, B) S 2p, (C, D) Ti 2p and (E, F) Si 2p for TiS_2_/LSPSC composite electrode in Li-In|LSPSC|TiS_2_ cell before and after cycling (fully charged state) 1000 cycles at 0.076 mA cm^-2^ and 30℃.


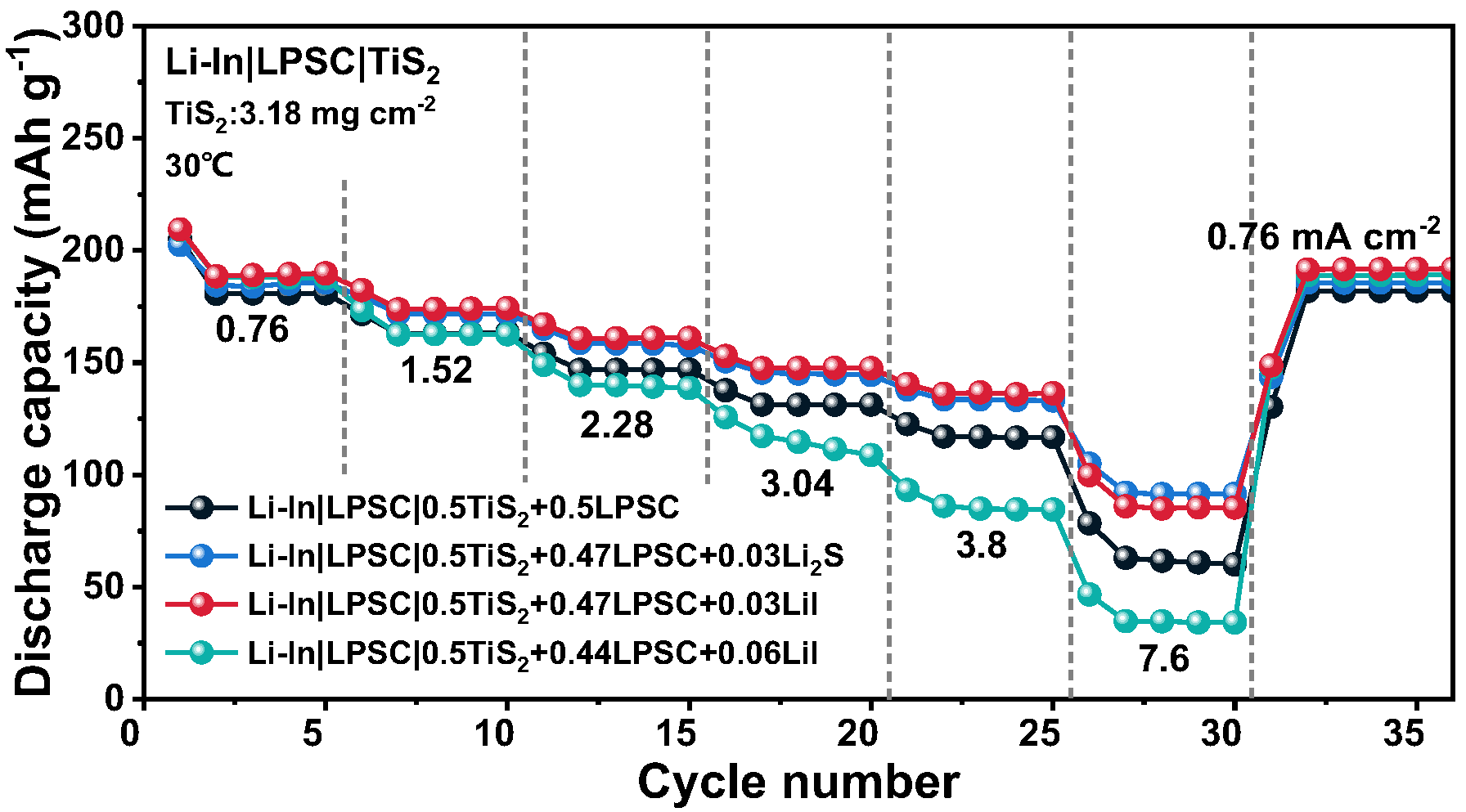


**Supplementary Figure 15.** Rate capability at 30℃ of TiS_2_/LPSC composite cathodes without and with Li_2_S/LiI additive, including 0.5TiS_2_+0.5LPSC (black), 0.5TiS_2_+0.47LPSC+0.03Li_2_S (blue), 0.5TiS_2_+0.47LPSC+0.03LiI (red) and 0.5TiS_2_+0.44LPSC+0.06LiI (cyan).


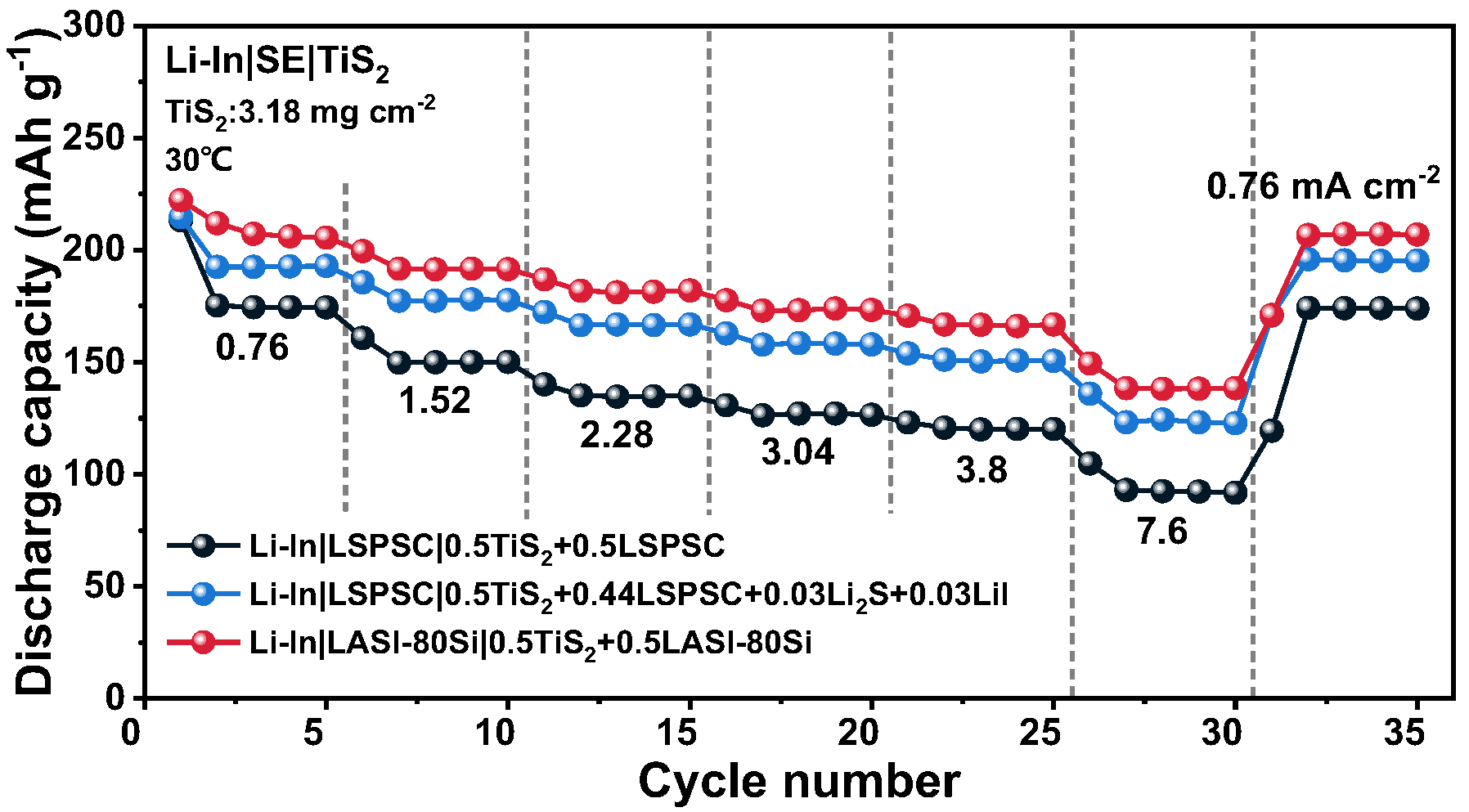


**Supplementary Figure 16.** Rate capability at 30℃ of TiS_2_ composite cathodes without and with Li_2_S/LiI additive, including 0.5TiS_2_+0.5LSPSC (black), 0.5TiS_2_+0.44LSPSC+0.03Li_2_S+0.03LiI (blue), and 0.5TiS_2_+0.5LASI-80Si (red).


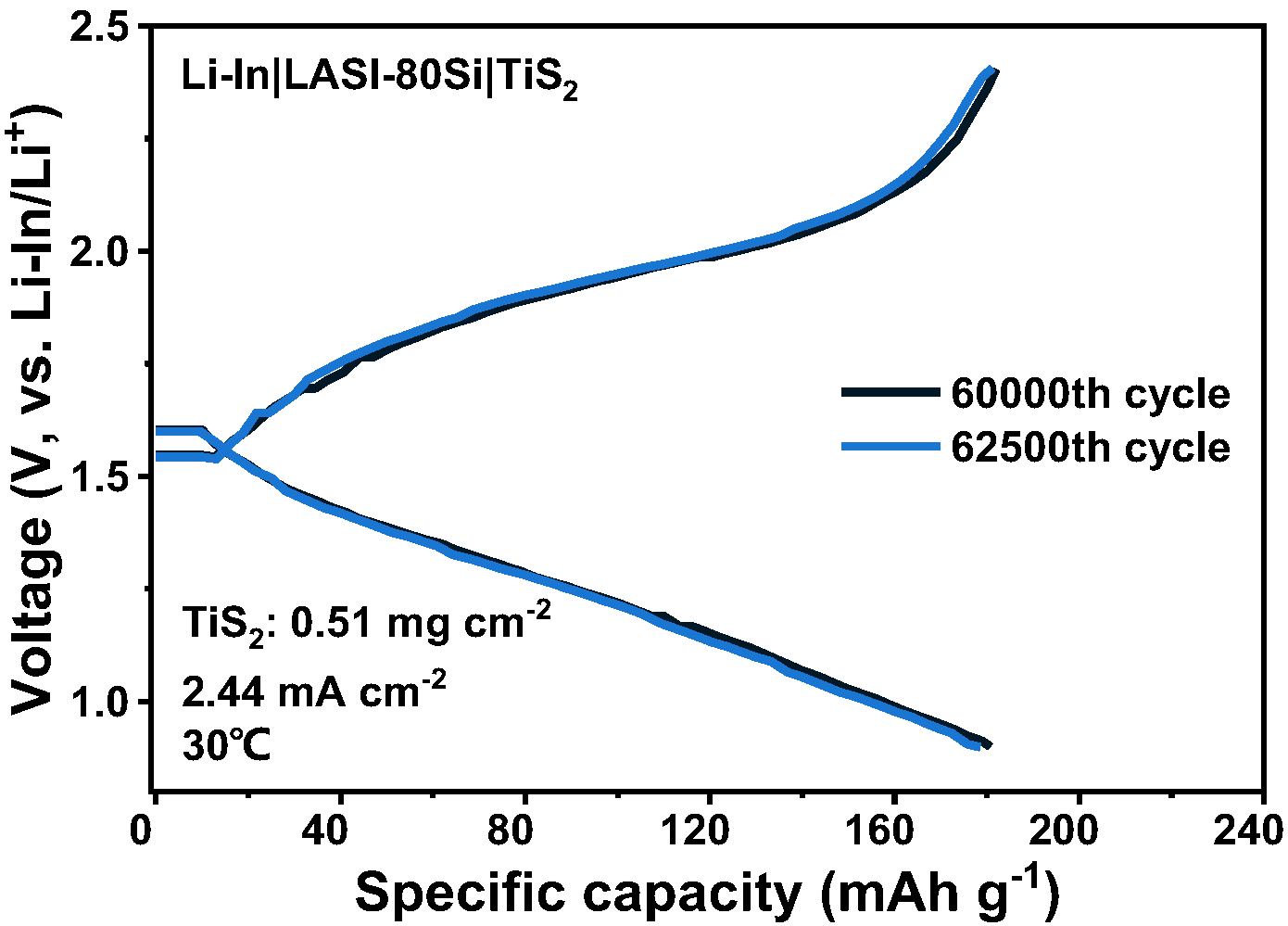


**Supplementary Figure 17.** The selected discharge-charge curves of Li-In|LASI-80Si| TiS_2_ ASSB at 2.44 mA cm^-2^ and 30 ℃.

**Supplementary Table 1.** The ionic and electronic conductivity at 25 ℃ of LPSI, LPSC, LSPSC and LASI-80Si, calculated from the total resistance, thickness and area (0.7854 cm^2^) of the cold-pressed pellets.

| Electrolytes | Thickness (cm) | Total resistance (Ω) | Ionic conductivity (S/cm) | Electronic conductivity (S/cm) |
| --- | --- | --- | --- | --- |
| LPSI | 0.0609 | 23300 | 3.33 × 10^-6^ | 2.32 × 10^-9^ |
| LPSC | 0.0674 | 22.5 | 3.81 × 10^-3^ | 3.94 × 10^-9^ |
| LSPSC | 0.0693 | 8.7 | 1.01 × 10^-2^ | 1.32× 10^-8^ |
| LASI-80Si | 0.0637 | 7.8 | 1.04 × 10^-2^ | 5.03 × 10^-9^ |

**Supplementary Table 2.** The peak position, intensity and intensity ratio of (311) and (222) crystal planes (denoted by A and B, respectively) for LASI, LASI-ySn (y = 10, 30) and LASI-ySi (y = 20, 80).

| Electrolytes | Substitution proportion y | Position A | Intensity I_A_ | Position B | Intensity I_B_ | I_A_/I_B_ |
| --- | --- | --- | --- | --- | --- | --- |
| LASI | 0 | 28.91 | 3389 | 30.20 | 4167 | 0.8132 |
| LASI-ySn | 10 | 28.85 | 3305 | 30.16 | 3417 | 0.9672 |
|  | 30 | 28.79 | 2264 | 30.11 | 2486 | 0.9107 |
| LASI-ySi | 20 | 28.96 | 3097 | 30.25 | 3041 | 1.0184 |
|  | 80 | 28.98 | 4875 | 30.27 | 2639 | 1.8472 |

**Supplementary Table 3.** The ionic conductivity at 25 ℃ calculated from the total resistance, thickness and area (0.7854 cm^2^) of the cold-pressed pellets of Li_6+x_Sn_x_As_1–x_S_5_I (x=0, 0.05, 0.10, 0.15, 0.20, 0.30, 0.40, 0.60) denoted as LASI-ySn (x = y %) and Li_6+x_Si_x_As_1–x_S_5_I (x=0, 0.10, 0.20, 0.30, 0.40, 0.50, 0.60, 0.70, 0.80, 0.90, 1.00) denoted as LASI-ySi (x = y %), and the geometric density deduced from the mass (0.1 g), thickness and area of pellets.

| Electrolytes | Substitution proportion x | Thickness (cm) | Total resistance (Ω) | Ionic conductivity (S cm^-1^) | Geometric density (g cm^-3^) |
| --- | --- | --- | --- | --- | --- |
| LASI | 0 | 0.0551 | 17900 | 3.92 × 10^-6^ | 2.311 |
| LASI-xSn | 5 | 0.0617 | 3940 | 1.99 × 10^-5^ | 2.064 |
|  | 10 | 0.0514 | 798 | 8.20 × 10^-5^ | 2.477 |
|  | 15 | 0.0527 | 336 | 2.00 × 10^-4^ | 2.416 |
|  | 20 | 0.0533 | 344 | 1.97 × 10^-4^ | 2.389 |
|  | 30 | 0.0546 | 347 | 2.00 × 10^-4^ | 2.332 |
|  | 40 | 0.0535 | 816 | 8.35 × 10^-5^ | 2.380 |
|  | 60 | 0.0518 | 1080 | 6.11 × 10^-5^ | 2.458 |
| LASI-xSi | 10 | 0.0558 | 8770 | 8.10 × 10^-6^ | 2.282 |
|  | 20 | 0.0550 | 2480 | 2.82 × 10^-5^ | 2.315 |
|  | 30 | 0.0594 | 89.9 | 8.41 × 10^-4^ | 2.143 |
|  | 40 | 0.0604 | 22.1 | 3.48 × 10^-3^ | 2.108 |
|  | 50 | 0.0611 | 11.4 | 6.82 × 10^-3^ | 2.084 |
|  | 60 | 0.0618 | 12.3 | 6.40 × 10^-3^ | 2.060 |
|  | 70 | 0.0612 | 9.62 | 8.10 × 10^-3^ | 2.080 |
|  | 80 | 0.0637 | 7.8 | 1.04 × 10^-2^ | 1.999 |
|  | 90 | 0.0624 | 42.1 | 1.89 × 10^-3^ | 2.040 |
|  | 100 | 0.0613 | 81700 | 9.55 × 10^-7^ | 2.077 |

**Supplementary Table 4.** The cold-pressed ionic conductivity at 25 ℃ and activation energy of representative sulfide SEs in each category.

| Classification | Electrolyte | Cold-pressed ionic conductivity (mS cm^-1^) | Activation energy (eV) | Ref. |
| --- | --- | --- | --- | --- |
| Glass | Li_2_S-P_2_S_5_-LiI | 2 | 0.305 | ^1^ |
| Glass-ceramics | Li_7_P_3_S_11_ | 3.2 | 0.177 | ^2^ |
| Thio-LISCON | Li_3.25_Ge_0.25_P_0.75_S_4_ | 2.2 | 0.208 | ^3^ |
| LGPS family | Li_10_GeP_2_S_12_ | 9 | 0.25 | ^4^ |
|  | Li_9.54_Si_1.74_P_1.44_S_11.7_Cl_0.3_ | 16 | 0.24 | ^5^ |
| Argyrodites | Li_5.5_PS_4.5_Cl_1.5_ | 10.2 | 0.27 | ^6^ |
|  | Li_6.6_Ge_0.6_P_0.4_S_5_I | 5.4 | 0.24 | ^7^ |
|  | Li_6.6_Ge_0.5_Sb_0.5_S_5_I | 16.1 | 0.18 | ^8^ |
|  | Li_6.8_Si_0.8_As_0.2_S_5_I | 10.4 | 0.20 | ^9^ |

**Supplementary Table 5.** Lattice parameter, fractional atomic coordinates, site occupancies and isotropic atomic displacement parameters (Uiso) of representative Li_6.8_Si_0.8_As_0.2_S_5_I obtained from X-ray powder diffraction at 300K.

| Space group $F\bar{4}3m$, a = 10.2100(2) Å, wRp = 11.75%, Rp = 8.92%, χ^2^ = 4.445,  94.87 wt% Li_6.8_Si_0.8_As_0.2_S_5_I, 2.64 wt% LiI, 2.49 wt% Li_2_S | | | | | | |
| --- | --- | --- | --- | --- | --- | --- |
| Atom | Fractional coordinates | | | Wyckoff sites | Occ. | Uiso (Å^2^) |
|  | x | y | z |  |  |  |
| Li | 0.2076(4) | 0.2076(4) | 0.0182(9) | 48h | 0.870(5) | 0.0925(5) |
| As | 0.5 | 0.5 | 0.5 | 4b | 0.182(4) | 0.0130(6) |
| Si | 0.5 | 0.5 | 0.5 | 4b | 0.817(6) | 0.0130(6) |
| S1 | 0.25 | 0.25 | 0.25 | 4c | 0.990(1) | 0.0197(3) |
| S2 | 0.6216(4) | 0.6216(4) | 0.6216(4) | 16e | 1 | 0.0243(3) |
| S3 | 0 | 0 | 0 | 4a | 0.083(8) | 0.0413(8) |
| I1 | 0 | 0 | 0 | 4a | 0.916(2) | 0.0413(8) |
| I2 | 0.25 | 0.25 | 0.25 | 4c | 0.009(9) | 0.0197(3) |

**Supplementary Table 6.** The weight percentage of all elements contained in Li_6.8_Si_0.8_As_0.2_S_5_I determined by chemical analysis method.

| Method | Element | Weight (%) | Theoretical weight (%) | Measurement error (%) |
| --- | --- | --- | --- | --- |
| ICP-AES | Li | 12.5 | 12.7 | 1.57 |
|  | Si | 5.9 | 6.0 | 1.67 |
|  | As | 4.0 | 4.0 | 0 |
| Carbon sulfur analysis | S | 43.3 | 43.1 | 0.46 |
| EDS mapping | I | 35.84 | 34.1 | 5.1 |

**Supplementary Table 7.** Root-mean square errors of the energy (meV/atom) and force (meV/Å) of LASI and LASI-80Si on the whole dataset generated from DP-Gen scheme.

| Root-mean square errors | LASI | LASI-80Si |
| --- | --- | --- |
| Energy (meV/atom) | 1.06 | 1.36 |
| Force (meV/Å) | 38.8 | 45.6 |

**Supplementary Table 8.** Exploration settings of DP-Gen iterations for LASI

| **Iteration** | **Temperature** | **Press (bar)** | **MD steps** | **Ensemble** | **New data generated (frames)** |
| --- | --- | --- | --- | --- | --- |
| 1 | 500, 700, 900 | - | 10000 | NVT | 208 |
| 2 | 500, 700, 900 | 0, 1.0 | 20000 | NPT | 151 |
| 3 | 700, 900, 1000 | 1.0, 10.0 | 200000 | NPT | 400 |
| 4 | 700, 900, 1000 | - | 800000 | NVT | 400 |
| 5 | 700, 900, 1000 | 1.0, 10.0 | 1000000 | NPT | 132 |

**Supplementary Table 9.** Exploration settings of DP-Gen iterations for LASI-80Si

| **Iteration** | **Temperature (K)** | **Press (bar)** | **MD steps** | **Ensemble** | **New data generated (frames)** |
| --- | --- | --- | --- | --- | --- |
| 1 | 500, 700, 900 | - | 500000 | NVT | 467 |
| 2 | 700, 900, 1000 | 0, 1.0 | 1000000 | NPT | 3200 |
| 3 | 700, 900, 1000 | 1.0, 10.0 | 1000000 | NPT | 3200 |
| 4 | 800, 900, 1000 | 1.0, 10.0 | 2000000 | NPT | 344 |
| 5 | 800, 900, 1000 | - | 1000000 | NVT | 256 |
| 6 | 800, 900, 1000 | 0.0, 1.0, 10.0 | 2000000 | NPT | 41 |

**Supplementary Table 10.** Fractional atomic coordinates, site occupancies of the optimal structure of Li_6_AsS_5_I (LASI) with computed lattice constant a = 10.458 Å at 400 K.

| **Atom** | **Fractional coordinates** | | | **Wyckoff sites** | **Occ.** |
| --- | --- | --- | --- | --- | --- |
|  | **x** | **y** | **z** |  |  |
| Li1 | 0.197 | 0.197 | 0.021 | 48h | 0.305 |
| Li2 | 0.021 | 0.25 | 0.25 | 24g | 0.39 |
| Li3 | 0.228 | 0.085 | 0.085 | 48h | 0.001 |
| Li4 | 0.857 | 0.143 | 0.143 | 16e | 0 |
| As | 0.5 | 0.5 | 0.5 | 4b | 1 |
| I | 0 | 0 | 0 | 4a | 1 |
| S1 | 0.621 | 0.621 | 0.621 | 16e | 1 |
| S2 | 0.25 | 0.25 | 0.25 | 4c | 1 |

**Supplementary Table 11.** Fractional atomic coordinates, site occupancies of the optimal structure of Li_6.8_Si_0.8_As_0.2_S_5_I (LASI-80Si) with computed lattice constant a = 10.414 Å at 400K.

| **Atom** | **Fractional coordinates** | | | **Wyckoff sites** | **Occ.** |
| --- | --- | --- | --- | --- | --- |
|  | **x** | **y** | **z** |  |  |
| Li1 | 0.216 | 0.216 | 0.016 | 48h | 0.297 |
| Li2 | 0.016 | 0.25 | 0.25 | 24g | 0.406 |
| Li3 | 0.229 | 0.073 | 0.073 | 48h | 0.064 |
| Li4 | 0.857 | 0.143 | 0.143 | 16e | 0.01 |
| As | 0.5 | 0.5 | 0.5 | 4b | 0.2 |
| Si | 0.5 | 0.5 | 0.5 | 4b | 0.8 |
| I | 0 | 0 | 0 | 4a | 1 |
| S1 | 0.618 | 0.618 | 0.618 | 16e | 1 |
| S2 | 0.251 | 0.251 | 0.25 | 4c | 1 |

**Supplementary Table 12.** The calculated diffusion coefficient and ionic conductivity of LASI at corresponding temperature.

| **LASI** | | |
| --- | --- | --- |
| **Temperature (K)** | **Diffusion coefficient (cm^2^ s^-1^)** | **Li^+^ conductivity (S cm^-1^)** |
| 550 | 2.12E-08 | 1.50E-03 |
| 600 | 5.52E-08 | 3.55E-03 |
| 650 | 1.82E-07 | 1.07E-02 |
| 700 | 5.07E-07 | 2.76E-02 |
| 800 | 1.74E-06 | 8.25E-02 |
| 1000 | 1.54E-05 | 5.67E-01 |

**Supplementary Table 13.** The calculated diffusion coefficient and ionic conductivity of LASI-25Si at corresponding temperature.

| **LASI-25Si** | | |
| --- | --- | --- |
| **Temperature (K)** | **Diffusion coefficient (cm^2^ s^-1^)** | **Li^+^ conductivity (S cm^-1^)** |
| 350 | 3.25E-07 | 3.79E-02 |
| 400 | 6.33E-07 | 6.47E-02 |
| 450 | 1.04E-06 | 9.36E-02 |
| 500 | 1.55E-06 | 1.27E-01 |
| 600 | 3.07E-06 | 2.06E-01 |

**Supplementary Table 14.** The calculated diffusion coefficient and ionic conductivity of LASI-50Si at corresponding temperature.

| **LASI-50Si** | | |
| --- | --- | --- |
| **Temperature (K)** | **Diffusion coefficient (cm^2^ s^-1^)** | **Li^+^ conductivity (S cm^-1^)** |
| 320 | 3.84E-07 | 5.14E-02 |
| 350 | 6.24E-07 | 7.62E-02 |
| 400 | 1.21E-06 | 1.29E-01 |
| 500 | 3.03E-06 | 2.56E-01 |
| 600 | 6.41E-06 | 4.50E-01 |

**Supplementary Table 15.** The calculated diffusion coefficient and ionic conductivity of LASI-80Si at corresponding temperature.

| **LASI-80Si** | | |
| --- | --- | --- |
| **Temperature (K)** | **Diffusion coefficient (cm^2^ s^-1^)** | **Li^+^ conductivity (S cm^-1^)** |
| 300 | 2.97E-07 | 4.45E-02 |
| 310 | 3.55E-07 | 5.15E-02 |
| 330 | 5.38E-07 | 7.30E-02 |
| 340 | 6.46E-07 | 8.53E-02 |
| 350 | 7.42E-07 | 9.53E-02 |
| 370 | 9.80E-07 | 1.19E-01 |
| 400 | 1.61E-06 | 1.80E-01 |
| 500 | 4.21E-06 | 3.73E-01 |
| 600 | 8.19E-06 | 5.32E-01 |

**Supplementary Table 16.** The calculated activation energy of pristine and Si-substituted LASI.

| **Sulfide SE** | **Activation energy (eV)** |
| --- | --- |
| LASI | 0.694 |
| LASI-25Si | 0.16 |
| LASI-50Si | 0.164 |
| LASI-80Si | 0.168 |

**Supplementary Table 17.** The total generation amount of H_2_S, the peak value of the generation rate and its corresponding peak position of LPSI, LPSC, LSPSC, LASI and LASI-80Si sulfide SEs, measured at 23%~25% RH and 26~29 ℃.

| Electrolyte | Total amount of H_2_S (cm^3^ g^-1^) | Peak value of the generation rate (cm^3^ g^-1^ min^-1^) | Peak position (min) |
| --- | --- | --- | --- |
| LPSI | 105.35 | 25.56 | 0.3333 |
| LPSC | 98.99 | 49.95 | 0.4167 |
| LASI | 75.07 | 37.19 | 0.4167 |
| LASI-80Si | 91.32 | 39.89 | 0.4167 |

**Supplementary Table 18.** The intensity variation of the strongest diffraction peak and the identified reaction products.

| **Electrolytes** | **Exposure time (min)** | **Peak (°)** | **Intensity (a.u.)** | **Structural retention rate (%)** | **Reaction products** |
| --- | --- | --- | --- | --- | --- |
| LPSI | 0 (pristine) | 24.95 | 4236 | 100.00 |  |
|  | 5 | 24.94 | 2708 | 63.93 | LiI·3H_2_O, Li_3_PO_4_ |
|  | 10 | 24.95 | 2223 | 52.48 |  |
|  | 20 | 25.15 | 722 | 17.04 |  |
| LPSC | 0 (pristine) | 30.04 | 4736 | 100.00 |  |
|  | 5 | 30.02 | 4278 | 90.33 | LiOH, LiOH·H_2_O |
|  | 10 | 30.03 | 4000 | 84.46 |  |
|  | 20 | 30.15 | 2833 | 59.82 |  |
| LSPSC | 0 (pristine) | 29.56 | 3612 | 100.00 |  |
|  | 5 | 29.59 | 2264 | 62.68 | Amorphization |
|  | 10 | 29.56 | 2028 | 56.15 |  |
|  | 20 | 29.63 | 1444 | 39.98 |  |
| LASI-80Si | 0 (pristine) | 24.62 | 3847 | 100.00 |  |
|  | 5 | 24.61 | 3514 | 91.34 | LiI·H_2_O |
|  | 10 | 24.66 | 3014 | 78.35 |  |
|  | 20 | 24.80 | 2042 | 53.08 |  |

**Supplementary Table 19.** The reduction peak, integral reduction specific current, oxidation peak, and integral oxidation specific current of Li|SE|SE/C cells with LPSI, LPSC, LSPSC, LASI-80Si sulfide SEs, measure at 30 ℃.

| Electrolyte | Reduction peak (V) | Integral reduction specific current (VA g^-1^) | Oxidation peak (V) | Integral oxidation specific current (VA g^-1^) |
| --- | --- | --- | --- | --- |
| LPSC | 2.278 | 0.01014 | 3.487 | 0.00297 |
| LSPSC | 2.311 | 0.05877 | 3.223 | 0.00246 |
| LASI-80Si | 2.227 | 0.01116 | 2.761 | 0.01046 |

**Supplementary Table 20.** The specific discharge and charge capacity, initial coulombic efficiency of Li-In|SE|TiS_2_ ASSBs with LPSI, LPSC, LSPSC and LASI-80Si sulfide SEs, respectively, at 30 ℃ and 0.076 mA cm^-2^.

| Cell configuration | Initial discharge capacity (mAh g^-1^) | Initial charge capacity (mAh g^-1^) | Initial Coulombic efficiency (%) |
| --- | --- | --- | --- |
| Li-In\|LPSI\|TiS_2_ (0.015 mA cm^-2^) | 69.2 | 32 | 46.24 |
| Li-In\|LPSC\|TiS_2_ | 224.2 | 211.8 | 94.48 |
| Li-In\|LSPSC\|TiS_2_ | 221.2 | 205 | 92.67 |
| Li-In\|LASI-80Si\|TiS_2_ | 227.9 | 228.7 | 100.33 |

**Supplementary Table 21.** The specific discharge and charge capacity, initial coulombic efficiency and capacity retention based on the second cycle after 1000 cycles for Li-In|SE|TiS_2_ ASSBs with LPSI, LPSC, LSPSC and LASI-80Si sulfide SEs, respectively, at 30 ℃ and 0.76 mA cm^-2^.

| Cell configuration | Initial discharge capacity (mAh g^-1^) | Initial charge capacity (mAh g^-1^) | Initial Coulombic efficiency (%) | Discharge capacity at 2nd cycle (mAh g^-1^) | Discharge capacity at 1000th cycle (mAh g^-1^) | Capacity retention after 1000 cycles |
| --- | --- | --- | --- | --- | --- | --- |
| Li-In\|LPSI\|TiS_2_ (0.015 mA cm^-2^) | 69.2 | 32 | 46.24 | - | - | - |
| Li-In\|LPSC\|TiS_2_ | 206.9 | 173.1 | 83.67 | 175.4 | 122.2 (768 cycles) | 69.67 (768 cycles) |
| Li-In\|LSPSC\|TiS_2_ | 213.2 | 171.4 | 80.39 | 175.3 | 148.8 | 84.88 |
| Li-In\|LASI-80Si\|TiS_2_ | 222.3 | 216.9 | 97.57 | 214.3 | 172 | 80.26 |

**Supplementary Table 22.** The first-cycle specific discharge and charge capacity, initial coulombic efficiency and rate capability for TiS_2_ composite cathodes without and with Li_2_S/LiI additive, including 0.5TiS_2_+0.5LPSC (black), 0.5TiS_2_+0.47LPSC+0.03Li_2_S (blue), 0.5TiS_2_+0.47LPSC+0.03LiI (red) and 0.5TiS_2_+0.47LPSC+0.06LiI (cyan), tested at 30 ℃.

| Cell configuration | Initial discharge capacity (mAh g^-1^) | Initial charge capacity (mAh g^-1^) | Initial Coulombic efficiency (%) | Discharge capacity at 3.8 mA cm^-2^ (mAh g^-1^) | Discharge capacity at 7.6 mA cm^-2^ (mAh g^-1^) |
| --- | --- | --- | --- | --- | --- |
| Li-In\|LPSC\|0.5TiS_2_+0.5LPSC | 205.2 | 179.3 | 87.38 | 117.1 | 63 |
| Li-In\|LPSC\|0.5TiS_2_+0.47LPSC+0.03Li_2_S | 202.4 | 182.7 | 90.26 | 133.6 | 92 |
| Li-In\|LPSC\|0.5TiS_2_+0.47LPSC+0.03LiI | 209.4 | 185.2 | 88.44 | 136.1 | 86 |
| Li-In\|LPSC\|0.5TiS_2_+0.47LPSC+0.06LiI | 209.1 | 183.1 | 87.56 | 86.1 | 34.8 |

**Supplementary Table 23.** The first-cycle specific discharge and charge capacity, initial coulombic efficiency and rate capability for TiS_2_ composite cathodes without and with Li_2_S and LiI additive, including 0.5TiS_2_+0.5LSPSC (black), 0.5TiS_2_+0.44LSPSC+0.03Li_2_S+0.03LiI (blue), and 0.5TiS_2_+0.5LASI-80Si (red), tested at 30 ℃.

| Cell configuration | Initial discharge capacity (mAh g^-1^) | Initial charge capacity (mAh g^-1^) | Initial Coulombic efficiency (%) | Discharge capacity at 3.8 mA cm^-2^ (mAh g^-1^) | Discharge capacity at 7.6 mA cm^-2^ (mAh g^-1^) |
| --- | --- | --- | --- | --- | --- |
| Li-In\|LSPSC\|0.5TiS_2_+0.5LSPSC | 213.2 | 171.4 | 80.39 | 120.8 | 93 |
| Li-In\|LPSC\|0.5TiS_2_+0.44LPSC+0.03Li_2_S+0.03LiI | 214.7 | 191.5 | 89.19 | 151.1 | 123.2 |
| Li-In\|LASI-80Si\|0.5TiS_2_+0.5LASI-80Si | 224.4 | 222.3 | 99.06 | 166.8 | 138.4 |

**Supplementary Table 24.** The comparison of long-term cyclability of ASSBs with TiS_2_ cathode and various solid electrolytes.

| No. | Cell configuration | Current density (mA cm^-2^) | Mass loading (weight content) of active materials (mg cm^-2^) | Operating pressure (MPa) | Reversible capacity (mAh g^-1^) | Working temperature (℃) | Cycle number (cycles) | Capacity retention (%) | Ref. |
| --- | --- | --- | --- | --- | --- | --- | --- | --- | --- |
| 1 | Li\|Li_2_B_12_H_12_\|TiS_2_ | 0.0455 | 3.98 (40%) | - | 228 | 80 | 20 | 81.58 | ^10^ |
| 2 | Li-In\|0.8Li_2_S-0.2P_2_S_5_\|0.25TiS_2_-0.75LiN | 0.044 | 2.51 (33.3%) | - | 205 | 25 (RT) | 60 | 78 | ^11^ |
| 3 | Li\|0.47LiBH_4_−0.53MgO \|TiS_2_ | 0.031 | 3.54 (50%) | - | 175 | 60 | 65 | > 80 | ^12^ |
| 4 | Li\|Li_2_(B_11_H_14_)(CB_11_H_12_)\|TiS_2_ | 0.04 | 0.82 (40%) | 3.2 | 188 | 60 | 150 | 82 | ^13^ |
| 5 | Li\|LiBH_4_ \|TiS_2_ | 0.196 | 4.78 (40%) | - | 205 | 120 | 300 | 88 | ^14^ |
| 6 | Li-In\|Li_6.8_Si_0.8_As_0.2_S_5_I\|TiS_2_ | 0.122 | 3.18 (50%) | 30 | 222.3 | 30 | 1000 | 80.26 | This work |
| 7 | Li-In\|Li_10_GeP_2_S_12_\|TiS_2_ | 0.87 | 3.68 (48.8%) | - | 215 | 30 | 50 | 68.5 | ^15^ |
| 8 | Li-In\|77.5Li_2_S-22.5P_2_S_5_\|TiS_2_ | 1.2 | 2.43 (32.3%) | - | 191 | 25 | 50 | 80.1 | ^16^ |
| 9 | Li-In\|Li_6_PS_5_Cl\|TiS_2_ | 0.78 | 23.1 (70%) |  | 228.1 | 60 | 100 | 76.9 | ^17^ |
| 10 | Li\|0.7Li(CB_9_H_10_)-0.3Li(CB_11_H_12_)\|TiS_2_ | 1.2 | 5.09 (40%) | 10 N m | 238.5 | 60 | 200 | 75.8 | ^18^ |
| 11 | Li-In\|Li_6_PS_5_Cl\|TiS_2_ | 1.95 | 23.1 (70%) | - | 187.8 | 60 | 500 | 72.9 | ^17^ |
| 12 | Li-In\|Li_10_GeP_2_S_12_\|TiS_2_ | 3 | 3.82 (30%) | 230 | 200 | 25 | 20 | 87.5 | ^19^ |
| 13 | Li-In\|77.5Li_2_S-22.5P_2_S_5_\|TiS_2_ | 6 | 2.43 (32.3%) | - | 208 | 60 | 50 | 72.11 | ^16^ |
| 14 | Li-In\|Li_6_PS_5_Cl\|TiS_2_ | 3.9 | 23.1 (70%) | - | 189.7 | 60 | 300 | 62.4 | ^17^ |
| 15 | Li-In\|Li_6.8_Si_0.8_As_0.2_S_5_I\|TiS_2_ | 2.44 | 0.51 (50%) | 30 | 123 | 30 | 62500 | 147% | This work |

**Supplementary Table 25.** The rate capability tested at 30 ℃ of Li-In|SE|TiS_2_ ASSBs with LPSI, LPSC, LSPSC and LASI-80Si sulfide SEs, respectively. The steady-state discharge capacity at the second cycle for each current rate was selected.

| Cell configuration | Discharge capacity at 0.76 mA cm^-2^ (mAh g^-1^) | Discharge capacity at 3.8 mA cm^-2^ (mAh g^-1^) | Discharge capacity at 7.6 mA cm^-2^ (mAh g^-1^) | Discharge capacity returning back 0.76 mA cm^-2^ (mAh g^-1^) |
| --- | --- | --- | --- | --- |
| Li-In\|LPSC\|TiS_2_ | 175.4 | 94.2 | 38 | 175 |
| Li-In\|LSPSC\|TiS_2_ | 175.3 | 120.8 | 93 | 174.2 |
| Li-In\|LASI-80Si\|TiS_2_ | 211.9 | 166.8 | 138.4 | 206.7 |

**Supplementary Table 26.** The comparison of upper-limit current density tested for ASSBs or lithium-ion batteries with TiS_2_ cathode and various solid electrolytes (SEs) or organic liquid electrolytes (OLEs).

| No. | Cell configuration | Current density (mA cm^-2^) | Weight content of active materials (%) | Operating pressure (MPa) | Specific Capacity at corresponding rate (mAh g^-1^) | Working temperature (℃) | Ref. |
| --- | --- | --- | --- | --- | --- | --- | --- |
| 1 | Li\|LiPF_6_-EC/DMC\|TiS_2_ | 1.43 | 80 | - | 130 | - | ^20^ |
| 2 | Li-In\|Li_6.7_Si_0.7_Sb_0.3_S_5_I\|TiS_2_ | 1.52 | 50 | 187 | 152 | 30 | ^21^ |
| 3 | Li-In\|Li_3_PS_4_\|TiS_2_ | 2.4 | 50 | - | 109 | 30 | ^22^ |
| 4 | Li\|77.5Li_2_S-22.5P_2_S_5_\|Non-ball-milled TiS_2_ | 3.6 | 32.3 | - | 60 | 25 | ^16^ |
| 5 | Li\|77.5Li_2_S-22.5P_2_S_5_\|Small-ball-milled TiS_2_ | 3.6 | 32.3 | - | 80 | 25 | ^16^ |
| 6 | Li\|Li_6_PS_5_Cl\|TiS_2_ | 3.9 | 70 | - | ~ 175 | 60 | ^17^ |
| 7 | Li-In\|Li_10_GeP_2_S_12_\|TiS_2_ | 4.4 | 30 | 230 | ≤100 | - | ^19^ |
| 8 | Li\|77.5Li_2_S-22.5P_2_S_5_\|Non-ball-milled TiS_2_ | 6 | 32.3 | - | 20 | 60 | ^16^ |
| 9 | Li\|77.5Li_2_S-22.5P_2_S_5_\|Small-ball-milled TiS_2_ | 6 | 32.3 | - | 180 | 60 | ^16^ |
| 10 | Li-In\|Li_10_GeP_2_S_12_\|TiS_2_ | 12 | 50 | - | 68 | 30 | ^22^ |
| 11 | Li\|0.7Li-(CB_9_H_10_)-0.3Li(CB_11_H_12_)\|TiS_2_ | 12.16 | 40 | 10 (N m) | 170 | 60 | ^18^ |
| 12 | Li-In\|Li_3_PS_4_/Li_10_GeP_2_S_12_\|TiS_2_ (bulk) | 17.64 | 48.8 | - | 10 | 30 | ^15^ |
| 13 | Li-In\|Li_3_PS_4_/Li_10_GeP_2_S_12_\|TiS_2_(nanosheet) | 17.64 | 48.8 | - | 50 | 30 | ^15^ |
| 14 | Li-In\|Li_6.8_Si_0.8_As_0.2_S_5_I\|TiS_2_ | 24.45 | 50 | 30 | 26 | 30 | This work |

**Supplementary Table 27.** The comparison of areal mass loading and areal capacity of active materials for ASSBs with TiS_2_ cathode and various solid electrolytes.

| No. | Cell configuration | Areal mass loading (mg cm^-2^) | Areal capacity (mAh cm^-2^) | Weight content of active materials (%) | Operating pressure (MPa) | Specific capacity (mAh g^-1^) | Current density (mA cm^-2^) | Working temperature (℃) | Ref. |
| --- | --- | --- | --- | --- | --- | --- | --- | --- | --- |
| 1 | Li\|Li_2_(B_11_H_14_)(CB_11_H_12_)\|TiS_2_ | 0.82 | 0.2 | 40 | 3.2 | 190 | 0.04 | 60 | ^13^ |
| 2 | Li\|77.5Li_2_S-22.5P_2_S_5_\|Non-ball-milledTiS_2_ | 2.43 | 0.48 | 32.3 | - | 191 | 0.96 | 25 | ^16^ |
| 3 | Li\|77.5Li_2_S-22.5P_2_S_5_\|Small-ball-milled TiS_2_ | 2.43 | 0.56 | 32.3 | - | 223 | 0.96 | 25 | ^16^ |
| 4 | Li-In\|Li_3_PS_4_\|TiS_2_ | 3.77 | 0.87 | 50 | - | 232 | 0.174 | 30 | ^22^ |
| 5 | Li-In\|Li_10_GeP_2_S_12_\|TiS_2_ | 3.77 | 0.92 | 50 | - | 245 | 0.174 | 30 | ^22^ |
| 6 | Li\|0.47LiBH_4_−0.53MgO\|TiS_2_ | 3.54 | 0.62 | 50 | - | 175 | 0.05 | 60 | ^12^ |
| 7 | Li-In\|Li_10_GeP_2_S_12_\|TiS_2_ | 3.82 | 0.76 | 30 | 230 | 200 | 0.076 | 25 | ^19^ |
| 8 | Li\|Li_2_B_12_H_12_\|TiS_2_ | 3.98 | 0.91 | 40 | - | 230 | 0.0455 | 80 | ^10^ |
| 9 | Li\|LiBH_4_\|TiS_2_ | 4.77 | 0.98 | 40 | - | 205 | 0.23 | 120 | ^14^ |
| 10 | Li\|0.7Li(CB_9_H_10_)-0.3Li(CB_11_H_12_)\|TiS_2_ | 5.09 | 1.22 | 40 | 10 (N m) | 239 | 0.122 | 60 | ^18^ |
| 11 | Li\|Li_6_PS_5_Cl\|Ball-milled TiS_2_ | 23.1 | 4.59 | 70 | - | - | 2.31 | 25 | ^17^ |
| 12 | Li-In\|Li_6.7_Si_0.7_Sb_0.3_S_5_I\|TiS_2_ | 23.8 | 5.59 | 50 | 187 | 235 | 0.559 | 30 | ^21^ |
| 13 | Li-In\|Li_6.8_Si_0.8_As_0.2_S_5_I\|TiS_2_ | 31.83 | 7.19 | 50 | 30 | 225.9 | 0.719 | 30 | This work |
| 14 | Li-In\|Li_6.8_Si_0.8_As_0.2_S_5_I\|TiS_2_ | 44.56 | 9.26 | 50 | 30 | 207.6 | 0.926 | 30 | This work |

**Supplementary Table 28.** The specification of ASSBs fabricated and operated at 30 MPa and 30℃ in this work.

| Cell configuration | Mass loading of TiS_2_ （mg cm^-2^） | Theoretical (practical) areal capacity (mAh cm^-2^) | Gravimetric ration of TiS_2_:SE in cathode | Mass of SE (mg) | Mass ratio of Li:In | Test item |
| --- | --- | --- | --- | --- | --- | --- |
| Li-In\|Li_6_PS_5_I\|TiS_2_ | 3.18 | 0.76 | 50:50 | 100 | 1: 25 | Charge-discharge profiles |
| Li-In\|Li_6_PS_5_Cl\|TiS_2_ | 3.18 | 0.76 | 50:50 | 100 | 1: 25 | Charge-discharge profiles; long-term cycle stability at 0.76 mA cm^-2^; rate capability from 0.76 mA cm^-2^ to 7.6 mA cm^-2^ |
| Li-In\|Li_9.54_Si_1.74_P_1.44_S_11.7_Cl_0.3_\|TiS_2_ | 3.18 | 0.76 | 50:50 | 100 | 1: 25 | Charge-discharge profiles; long-term cycle stability at 0.76 mA cm^-2^; rate capability from 0.76 mA cm^-2^ to 7.6 mA cm^-2^ |
| Li-In\|Li_6.8_Si_0.8_As_0.2_S_5_I\|TiS_2_ | 3.18 | 0.76 | 50:50 | 100 | 1: 25 | Charge-discharge profiles; long-term cycle stability at 0.76 mA cm^-2^; rate capability from 0.76 mA cm^-2^ to 7.6 mA cm^-2^ |
| Li-In\|Li_6.8_Si_0.8_As_0.2_S_5_I\|TiS_2_ | 0.51 | 0.12 | 50:50 | 100 | 1:25 | long-term cycle stability at 2.445 mA cm^-2^ |
| Li-In\|Li_6.8_Si_0.8_As_0.2_S_5_I\|TiS_2_ | 0.51 | 0.12 | 50:50 | 100 | 1:25 | Rate capability from 0.122 to 24.45 mA cm^-2^ |
| Li-In\|Li_6.8_Si_0.8_As_0.2_S_5_I\|TiS_2_ | 31.83 | 7.64 (7.00) | 50:50 | 100 | 1:25 | High mass loading at 0.76 mA cm^-2^ |
| Li-In\|Li_6.8_Si_0.8_As_0.2_S_5_I\|TiS_2_ | 44.56 | 10.70 (9.22) | 70:30 | 100 | 1:25 | High mass loading at 0.53 and 1.06 mA cm^-2^ |

**Supplementary References**

1. R. Mercier J-PM, B. Fahys and G. Robert. Superionic Conduction in Li_2_S-P_2_S_5_-Lil-GlassES. *Solid State Ionics* **5**, 663-666 (1981).

2. Seino Y, Ota T, Takada K, Hayashi A, Tatsumisago M. A sulphide lithium super ion conductor is superior to liquid ion conductors for use in rechargeable batteries. *Energy Environ Sci* **7**, 627-631 (2014).

3. Kanno R, Murayama M. Lithium Ionic Conductor Thio-LISICON: The Li_2_S-GeS_2_-P_2_S_5_ System. *Journal of The Electrochemical Society* **148**, (2001).

4. Kamaya N*, et al.* A lithium superionic conductor. *Nat Mater* **10**, 682-686 (2011).

5. Yuki Kato SH, Toshiya Saito, Kota Suzuki, Masaaki Hirayama, Akio Mitsui, Masao Yonemura, Hideki Iba and Ryoji Kanno. High-power all-solid-state batteries using sulfide superionic conductors. *NATURE ENERGY* **1**, (2016).

6. Jung WD*, et al.* Superionic Halogen-Rich Li-Argyrodites Using In Situ Nanocrystal Nucleation and Rapid Crystal Growth. *Nano Letters* **20**, 2303-2309 (2020).

7. Kraft MA*, et al.* Inducing High Ionic Conductivity in the Lithium Superionic Argyrodites Li6+ xP1- xGe xS5I for All-Solid-State Batteries. *J Am Chem Soc* **140**, 16330-16339 (2018).

8. Lee Y*, et al.* Lithium Argyrodite Sulfide Electrolytes with High Ionic Conductivity and Air Stability for All-Solid-State Li-Ion Batteries. *ACS Energy Letters*, 171-179 (2021).

9. Pushun Lu*, et al.* Wide‐Temperature, Long‐Cycling, and High‐Loading Pyrite All‐Solid‐State Batteries Enabled by Argyrodite Thioarsenate Superionic Conductor. *Advanced Functional Materials* **33**, 2211211 (2022).

10. Kim S*, et al.* Fast Lithium-Ion Conduction in Atom-Deficient closo-Type Complex Hydride Solid Electrolytes. *Chemistry of Materials* **30**, 386-391 (2018).

11. Yersak TA, Trevey JE, Lee S-H. In situ lithiation of TiS2 enabled by spontaneous decomposition of Li3N. *Journal of Power Sources* **196**, 9830-9834 (2011).

12. Gulino V*, et al.* Room-Temperature Solid-State Lithium-Ion Battery Using a LiBH4-MgO Composite Electrolyte. *ACS Appl Energy Mater* **4**, 1228-1236 (2021).

13. Payandeh S*, et al.* Nido‐Hydroborate‐Based Electrolytes for All‐Solid‐State Lithium Batteries. *Advanced Functional Materials* **31**, (2021).

14. Unemoto A*, et al.* Stable Interface Formation between TiS2 and LiBH4 in Bulk-Type All-Solid-State Lithium Batteries. *Chemistry of Materials* **27**, 5407-5416 (2015).

15. Oh DY*, et al.* All-solid-state lithium-ion batteries with TiS2 nanosheets and sulphide solid electrolytes. *Journal of Materials Chemistry A* **4**, 10329-10335 (2016).

16. Trevey JE, Stoldt CR, Lee S-H. High Power Nanocomposite TiS2 Cathodes for All-Solid-State Lithium Batteries. *Journal of The Electrochemical Society* **158**, (2011).

17. Kim JY*, et al.* Revisiting TiS2 as a diffusion-dependent cathode with promising energy density for all-solid-state lithium secondary batteries. *Energy Storage Materials* **41**, 289-296 (2021).

18. Kim S, Kisu K, Takagi S, Oguchi H, Orimo S-i. Complex Hydride Solid Electrolytes of the Li(CB_9_H_10_)–Li(CB_11_H_12_) Quasi-Binary System: Relationship between the Solid Solution and Phase Transition, and the Electrochemical Properties. *ACS Applied Energy Materials* **3**, 4831-4839 (2020).

19. Li WJ, Hirayama M, Suzuki K, Kanno R. Fabrication and All Solid-State Battery Performance of TiS2/Li10GeP2S12 Composite Electrodes. *Materials Transactions* **57**, 549-552 (2016).

20. Fleischmann S, Shao H, Taberna P-L, Rozier P, Simon P. Electrochemically Induced Deformation Determines the Rate of Lithium Intercalation in Bulk TiS2. *ACS Energy Letters* **6**, 4173-4178 (2021).

21. Zhou L, Assoud A, Zhang Q, Wu X, Nazar LF. New Family of Argyrodite Thioantimonate Lithium Superionic Conductors. *J Am Chem Soc* **141**, 19002-19013 (2019).

22. Shin BR, Nam YJ, Oh DY, Kim DH, Kim JW, Jung YS. Comparative Study of TiS 2 /Li-In All-Solid-State Lithium Batteries Using Glass-Ceramic Li 3 PS 4 and Li 10 GeP 2 S 12 Solid Electrolytes. *Electrochimica Acta* **146**, 395-402 (2014).
